# Human MAIT Cells Respond to *Staphylococcus aureus* with Enhanced Anti-Bacterial Activity

**DOI:** 10.3390/microorganisms10010148

**Published:** 2022-01-12

**Authors:** Andrew J. R. Cooper, Jonah Clegg, Féaron C. Cassidy, Andrew E. Hogan, Rachel M. McLoughlin

**Affiliations:** 1 Host Pathogen Interactions Group, School of Biochemistry and Immunology, Trinity Biomedical Sciences Institute, Trinity College Dublin, D02 PN40 Dublin, Ireland; cooperan@tcd.ie (A.J.R.C.); jonah.x.clegg@gsk.com (J.C.); 2 Kathleen Lonsdale Institute for Human Health Research, Maynooth University, W23 F2K8 Maynooth, Ireland; Fearon.Cassidy@mu.ie (F.C.C.); Andrew.E.Hogan@mu.ie (A.E.H.)

**Keywords:** *Staphylococcus aureus*, MAIT cell, vaccines, cell mediated immunity, IFNγ, dendritic cell

## Abstract

Mucosal-Associated Invariant T (MAIT) cells have been shown to play protective roles during infection with diverse pathogens through their propensity for rapid innate-like cytokine production and cytotoxicity. Among the potential applications for MAIT cells is to defend against *Staphylococcus aureus*, a pathogen of serious clinical significance. However, it is unknown how MAIT cell responses to *S. aureus* are elicited, nor has it been investigated whether MAIT cell cytotoxicity is mobilized against intracellular *S. aureus*. In this study, we investigate the capacity of human MAIT cells to respond directly to *S. aureus*. MAIT cells co-cultured with dendritic cells (DCs) infected with *S. aureus* rapidly upregulate CD69, express IFNγ and Granzyme B and degranulate. DC secretion of IL-12, but not IL-18, was implicated in this immune response, while TCR binding of MR1 is required to commence cytokine production. MAIT cell cytotoxicity resulted in apoptosis of *S. aureus*-infected cells, and reduced intracellular persistence of *S. aureus*. These findings implicate these unconventional T cells in important, rapid anti-*S. aureus* responses that may be of great relevance to the ongoing development of novel anti-*S. aureus* treatments.

## 1. Introduction

*Staphylococcus aureus* is a major cause of infection in diverse tissue settings, including the bloodstream where it is one of the most common and, unfortunately, most lethal, with mortality ranging from 15 to 50% [1,2,3]. These are striking statistics which are likely to be further exacerbated, given the rising problem of antibiotic-resistant strains [4]. There is an urgent need for new strategies to prevent and treat *S. aureus* infection. Despite significant efforts, an efficacious *S. aureus* vaccine remains elusive [5]. Multiple candidate vaccines have succeeded in inducing protective immunity in the mouse, but without successful translation to human subjects [6,7,8,9,10], highlighting the disparities that exist between murine and human immunity that are directly impeding progress within the field. It is worth noting that to date no candidate vaccine in late-stage efficacy trials has successfully mobilised enduring human T cell responses against *S. aureus* [11,12]. IFNγ and IL-17, key cytokines produced by T cells, are now known to be indispensable in anti-staphylococcal immunity. HIV, diabetes mellitus and end-stage renal disease, conditions that limit IFNγ production, are associated with heightened susceptibility to *S. aureus* bacteraemia [13,14], while Hyper-IgE syndrome, which leads to IL-17 deficiency, is associated with susceptibility to *S. aureus* mucosal infections [15,16]. While these patients typically have a number of risk factors that may predispose them to susceptibility, these correlations have inspired significant interest in how individual T cell subsets contribute to the protective immune response to *S. aureus*. T_h_1 and T_h_17 classes of helper T cells play important roles in activation of macrophages [17,18], neutrophil survival [19], and mediating the efficient killing of intracellular *S. aureus* by infected cells [20]. Naturally then, T cells are now considered important correlates of immunity for targeting in next-generation vaccines [21]. More recently a potential role for unconventional T cells, in particular γδ T cells, in *S. aureus* defence has emerged [22,23,24,25]. Importantly, γδ T cells have shown some hallmarks of pathogen-specific expansion and memory [26,27], suggesting the tantalizing possibility that these cells could be vaccine targetable [9].

One T cell population that has being largely overlooked in the quest to identify correlates of *S. aureus* immunity are the Mucosal-Associated Invariant T (MAIT) cells. A possible reason for their neglect may be their infrequency in murine tissues [28,29], but they contribute 1–10% of the T cells within human blood and are further enriched in barrier tissues [28,30]. MAIT cells are activated both by innate cytokines and by binding of the unique, conserved MAIT cell TCR to the MHC-like molecule MR1 in complex with the metabolites 5-OP-RU and 5-OE-RU, which are produced by a wide variety of bacteria but not human cells [31,32]. Once activated, MAIT cells are robust secretors of IFNγ and cytotoxic mediators in acute infection scenarios [33]. In mice, in spite of their low frequency, MAIT cells have been shown to play important non-redundant roles in protection against multiple pathogens. MR1-deficient mice (which lack MAIT cells) are susceptible to *Klebsiella pneumoniae* [34], *Mycobacterium bovis* [35], *Mycobacterium abscessus* [36], *Legionella longbeachae* [37], and polymicrobial sepsis [38]. In these studies, the absence of MAIT cells resulted in failure to control infection and in heightened mortality. Accumulating evidence also supports an important role for MAIT cells in human infection settings. Patients suffering from pulmonary infections like tuberculosis have lower circulating MAIT cell frequencies [36,39,40], and the distribution of *Mycobacterium*-reactive MAIT cells in TB-afflicted individuals suggests that peripheral MAIT cells traffic to the site of infection during active infection [36,39]. Severe cases of COVID-19 also correlate with reduced frequencies of circulating MAIT cells [40]. To date however there is relatively little understanding of how circulating MAIT cells contribute to immune defence within the bloodstream. Sepsis has been shown to be associated with a decrease in circulating MAIT cells [41], while MAIT cells from acute sepsis patients (at Day 1 post-admission) show notably modified phenotypic profiles compared to both MAIT cells from healthy donors, and MAIT cells from the same patients at Day 90 post-admission, suggesting acute MAIT cell responses which revert to the status quo ante following infection resolution; this includes significantly elevated expression of CD69 and reduced capacity for IFNγ production in response to *Escherichia coli* infection in vitro [38]. This study did not however distinguish data for specific causes of sepsis.

A role for MAIT cells in *S. aureus* bloodstream infection, protective or otherwise, has not yet been established. Notably, some specific MAIT cell clones can produce IFNγ in co-culture with *S. aureus*-infected cells [39], and when PBMCs are exposed to staphylococcal superantigens, MAIT cells are ‘hyperactivated’, resulting in strong IFNγ and TNF production, and eventual anergy; while a small part of this activation is accounted for by direct superantigen binding of the TCR, neutralisation of the innate cytokine IL-18 abolished the vast majority of cytokine production, demonstrating that MAIT cell responses to superantigens are largely cytokine-mediated and are secondary to the activation of other cells [42,43]. In addition to being robust secretors of IFNγ [33], a potentially important correlate of protection against *S. aureus* bloodstream infection in humans [18,44], MAIT cells are also strong secretors of cytotoxic mediators and play a role in the elimination of intracellular parasites [33]. Given *S. aureus*’s ability to survive intracellularly and even to disseminate inside circulating leukocytes [45,46,47], we might expect that cytotoxic responses to *S. aureus*-infected cells would be beneficial for combating *S. aureus* infection. However, the effects of MAIT cell cytotoxicity on *S. aureus* are completely untested. Additionally, no study has yet clarified the mechanism of activation of MAIT cells by *S. aureus*-infected cells, whether through MR1 presentation of riboflavin intermediates or through cytokines.

Here, we investigated the ability of human blood-derived MAIT cells to respond to *S. aureus*. MAIT cells responded to *S. aureus*-infected DCs with IFNγ and Granzyme B production. Both IL-12 and MR1 engagement of the MAIT TCR were implicated in this interaction. MAIT cells displayed an enhanced cytotoxic phenotype, with clear degranulation, which resulted in the apoptosis of *S. aureus*-infected cells, and reduced persistence of *S. aureus* in cultures.

## 2. Materials and Methods

### 2.1. Cell Culture

PBMCs were isolated by density gradient centrifugation using Ficoll (Lymphoprep). Monocytes were isolated from PBMCs by magnetic separation using a CD14 MACS kit (Miltenyi Biotec, North Rhine-Westphalia, Germany) and then cultured for propagation of DCs by incubation for 6 days in complete RPMI (RPMI 1640 (Sigma-Aldrich, Arklow, Ireland) with 10% heat-inactivated FBS (Sigma-Aldrich), 2 mM L-glutamine (Sigma-Aldrich), 100 U/mL penicillin (Sigma-Aldrich) and 100 µg/mL streptomycin (Sigma-Aldrich)) supplemented with 100 ng/mL GM-CSF (PeproTech, London, UK). On day 3, cells were supplemented with fresh complete RPMI formulated as above with an additional 50 ng/mL IL-4 (PeproTech). On day 6, monocyte-derived DCs were collected and resuspended in antibiotic-free media for infection. Cell purity was verified by flow cytometry by staining with CD11b and DC-SIGN and was routinely >95%.

MAIT cells were expanded in vitro as previously described [48]. Healthy donor PBMCs were cultured for 10–14 days in complete RPMI supplemented with 1 µg/mL 5-A-RU and 100 µM methylglyoxal, at a concentration of 1 × 10^6^ PBMCs/mL. On day 1200 µL of media from each well was discarded and replaced with 200 µL of fresh media containing IL-2 (6.75 ng/mL final concentration in culture). Following this, IL-2 was added by replacing 1 mL media (IL-2 concentration 33.3 ng/mL) every 3 days, and cells were monitored microscopically and split as needed. At day 10–14, cells were MACS-purified using APC-conjugated Abs against the MAIT cell TCR and an anti-APC MACS kit (Miltenyi Biotec), and tested for purity using fluorescently conjugated MR1 tetramers procured from the NIH tetramer facility. Purity was routinely >95%.

THP-1 cells were derived from maintained cell lines, obtained from the ECACC [49], cultured in complete RPMI and subcultured at 3 × 10^5^ cells/mL, approximately every 3 days.

### 2.2. Bacteria

*S. aureus* strains PS80 [50], USA300 LAC::*lux* [51] and Newman [52], and *E. coli* strain EC958 [53] have been described previously. *S aureus* strains were cultured overnight on Columbia agar supplemented with 2% NaCl (PS80), or on tryptic soy agar (USA300 and Newman); *E. coli* was grown overnight on tryptic soy agar supplemented with 4% defibrinated sheep’s blood (ThermoFisher Scientific, Gloucester, UK). Bacteria were suspended in PBS, enumerated by optical spectrometry and diluted to 1 × 10^8^ CFU/mL. CFU counts were verified by plating on appropriate agar overnight.

### 2.3. In Vitro Infection Assay

DCs were resuspended in antibiotic-free complete RPMI. Then, 1 × 10^5^ cells were transferred to each well of 96-well flat-bottom cell culture plates (Corning). DCs were inoculated with 1 × 10^6^ CFU of bacteria per well and incubated for 3 h before centrifugation and media replacement with complete RPMI supplemented with gentamicin (200 µg/mL; Sigma-Aldrich) to eliminate live extracellular bacteria. At this point, 1 × 10^5^ expanded MAIT cells were added to each well for the indicated lengths of time. For most assays (unless otherwise indicated), up to four sets of MAIT cells isolated from four independent donors were paired with each individual heterologous DC donor.

For specific experiments, neutralizing Abs for IL-12 p40 (clone C8.6, 20 µg/mL; Biolegend, San Diego, CA, USA), IL-18 (clone 914205, 20 µg/mL; R&D), or MR1 (clone 26.5, 10 µg/mL; Biolegend), or their isotype controls (IgG1κ and IgG2aκ; Biolegend; 10–20 µg/mL), were added to co-cultures, either alongside MAIT cells or, in the case of anti-MR1 antibodies, 30 min prior to addition of MAIT cells. For blocking of TCR activity, the small molecule inhibitor of tyrosine kinases Dasatinib [54] was added along with MAIT cells (100 nM) for the duration of culturing. For cell–cell contact-blocking experiments, DCs were cultured and infected in the lower chamber of a 96-well 0.4-mm transmembrane culture system (Millipore-Sigma, Arklow, Ireland) before gentamicin treatment as described, at which point MAIT cells were placed in the upper chamber.

For direct exposure of MAIT cells to bacteria, MAIT cells were resuspended in antibiotic-free complete RPMI and 1 × 10^5^ cells transferred to each well of 96-well flat-bottom cell culture plates (Corning, Flintshire, UK) before inoculation with 1 × 10^6^ CFU of bacteria per well. Cells were then incubated for 3 h before centrifugation, and media was replaced with complete RPMI supplemented with gentamicin (200 µg/mL; Sigma-Aldrich) to eliminate live extracellular bacteria.

THP-1 cells (1 × 10^5^ cells per well) were cultured in 96-well flat-bottom cell culture plates and infected with 1 × 10^5^ CFU of bacteria per well for 3 h before similar gentamicin treatment and addition of 1 × 10^5^ MAIT cells. At 24 h, THP-1 cells were collected to assess viability, using Annexin V/PI staining, and intracellular bacterial burdens.

In all experiments, supernatants were collected at specific timepoints for analysis of cytokine production by ELISA, and in some instances, cells were resuspended in media supplemented with brefeldin A (BFA) (10 µg/mL; Sigma-Aldrich) for the final 4 h of culture, after which cells were collected and stained for flow cytometry.

For CFU enumeration from infected cells, cultures were washed twice by centrifugation in sterile PBS before lysis for 10 min in 0.1% Triton X-100. Lysates were plated on TSA overnight before CFU enumeration.

### 2.4. Flow Cytometry

Following BFA treatment for the final 4 h, cells were resuspended in 1:1000 dilution of Fixable Viability Stain (Life Technologies, Gloucester, UK) for LIVE/DEAD cell determination. Cells were subsequently resuspended in PBS with 1% BSA (Sigma-Aldrich) and treated with Fcγ block (Life Technologies). For extracellular cell staining, cells were labelled with fluorochrome-conjugated Abs against CD3 (clone OKT3; Life Technologies), CD69 (clone FN50; BD Biosciences, Wokingham, UK), CD86 (clone IT2.2; Biolegend), DC-SIGN (clone eB-h209; Life Technologies) and CD107a (clone H4A3; Biolegend). Cells were then fixed and permeabilized using the FIX and PERM Kit (Life Technologies) before intracellular staining with fluorochrome-conjugated Abs against IFNγ (clone 4S.B3; Biolegend), IL-17A (clone eBIO64 DEC17; Life Technologies), Granzyme B (clone GB11; Biolegend), perforin (clone B-D48; Biolegend), granulysin (clone DH2; Biolegend), and granzyme A (clone CB9; Biolegend). For apoptosis analysis, cells were stained with Abs against Annexin V (Biolegend) and with propidium iodide (PI) (Biolegend) before immediate acquisition. Flow cytometric data were acquired with a BD LSRFortessa (BD Biosciences) and analysed using FlowJo v10.6.2 (Treestar, Ashland, OR, USA). Fluorescence minus one controls were used for the setting of gates.

### 2.5. ELISA

Sandwich ELISAs were performed on culture supernatants as per the manufacturer’s instructions for IFNγ, IL-12, IL-17A, TNF, Granzyme B (Biolegend) and IL-18 (Life Technologies).

### 2.6. Statistical Analysis

Statistical analysis was performed with SPSS Statistics 24 (IBM, New York, NY, USA) software. Friedman two-way ANOVA on groups was followed by pairwise Wilcoxon signed-rank post-tests. For direct comparisons of DC single cultures to cocultures in which DCs were generated from single donors and cocultured with MAIT cells from up to four separate donors, or comparisons of THP-1 cell single co-cultures to cocultures with MAIT cells from multiple donors, forward fill imputation was used to correctly match each DC or THP-1 value to each paired coculture value. In all situations, *p* ≤ 0.05 was considered significant.

## 3. Results

### 3.1. Human Blood-Derived MAIT Cells Are Activated in Co-Culture with S. aureus-Infected DCs

To establish if blood-derived MAIT cells could respond directly to *S. aureus*, MAIT cells were expanded in vitro from PBMCs. MR1 tetramer-positive cells were confirmed to be predominantly CD8^+^CD4^-^, with a smaller population of CD8^-^CD4^-^ cells and even fewer CD4^+^ cells (Figure S1A); these frequencies match those typically seen in MAIT cells isolated from the bloodstream [55], confirming that the process of in vitro expansion does not alter this population breakdown. All cells were CD161^+^, and a majority were CD161^hi^ (Figure S1C), suggestive of a largely mature cell population, again redolent of circulating MAIT cells [55].

DCs were infected with a panel of *S. aureus* isolates, in addition to the gram negative bacterium *E. coli*, a known activator of human MAIT cells [36]. Following 3 h infection, extracellular bacteria were eliminated from all cultures by gentamicin treatment, and MAIT cells were added for a further 24 h, after which cell culture supernatants were collected and cytokine secretion analysed by ELISA. Additionally, MAIT cells were directly exposed to live bacteria for 3 h, followed by gentamicin treatment, and supernatants collected 24 h post bacterial exposure. All cells were treated with Brefeldin A (BFA) for the final 4 h of culture, and subsequently cell populations analysed by flow cytometry.

**Figure 1:**
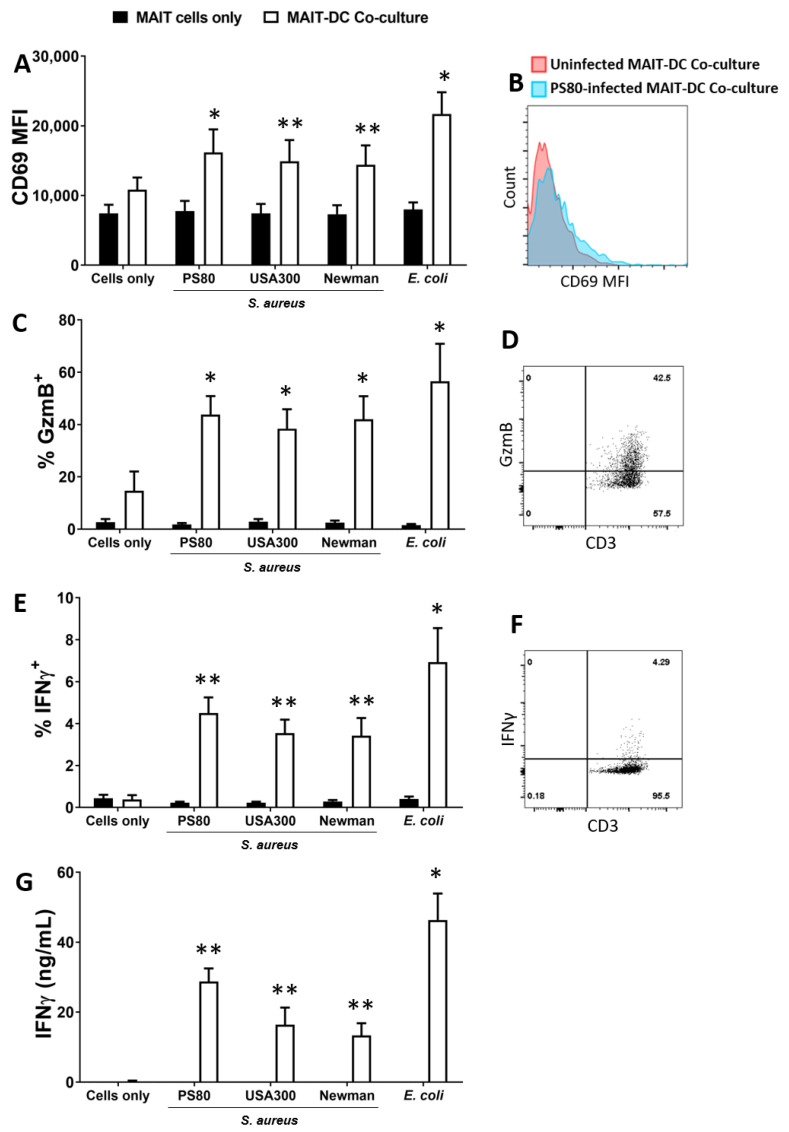

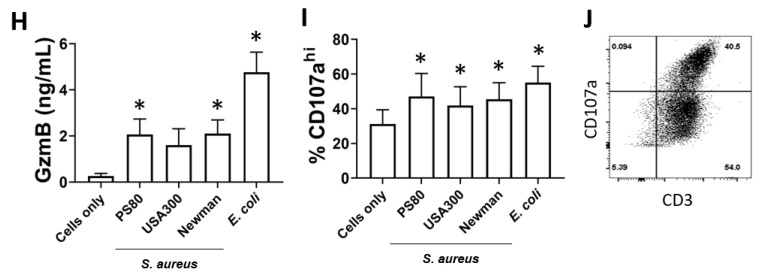
Human blood-derived Mucosal-Associated Invariant T (MAIT) cells are activated in co-culture with *S. aureus*-infected DCs to express CD69, Granzyme B, IFNγ and CD107a. MAIT cells and DCs (5 × 10^5^ cells/mL) were infected with *S. aureus* (strains PS80, USA300, Newman) and *E. coli* (strain EC958) at MOI 10 for 3 h before elimination of extracellular bacteria by gentamicin treatment. DCs were then co-cultured with uninfected MAIT cells (5 × 10^5^/mL) for 24 h. Cells were treated with BFA for the final 4 h of culture. Expression of CD69 (**A**), Granzyme B (‘GzmB’) (**C**), IFNγ (**E**) and CD107a (**I**) by MAIT cells were assessed by flow cytometry, and concentration of IFNγ (**G**) and Granzyme B (**H**) in culture supernatants assessed by ELISA. Results are expressed as mean fluorescence intensity (MFI) of total live singlet CD3^+^ cells + SEM (**A**), mean % positive cells within total live singlet CD3^+^ cells + SEM (**C**,**E**,**I**), or mean concentration in culture supernatants + SEM (**G**,**H**). Representative FACS plots for PS80 infection are shown (**B**,**D**,**F**,**J**). *n* = 4–10 DC donors per group, *n* = 7–18 MAIT cell donors per group. ‘Cells only’ refers to uninfected cultures. Statistical analysis by pairwise Wilcoxon signed rank test, with all columns compared directly to cells only. * *p* ≤ 0.05, ** *p* ≤ 0.01.

Direct exposure of MAIT cells to *S. aureus* did not result in expression of Granzyme B or IFNγ (Figure 1). By contrast, at 24 h MAIT cells had significantly upregulated CD69 (Figure 1A) and were producing high levels of both Granzyme B (Figure 1C) and IFNγ (Figure 1E,G) following co-culture with DCs infected with all *S. aureus strains*. Cytokine levels were comparable to those induced by *E. coli*-infected DCs. IL-17A was undetectable in all culture conditions tested, while TNF production in response to *S. aureus*-infected DCs was substantial in the co-cultures (Figure S2). MAIT cells also demonstrated significantly elevated expression of the degranulation marker CD107a (Figure 1H) and secretion of Granzyme B into culture media (Figure 1I) following co-culture with *S. aureus* or *E.coli*-infected DCs, suggesting a rapid adoption of a cytotoxic phenotype alongside the IFNγ response. In spite of high Granzyme expression, DC viability was not reduced in the co-cultures at 24 h (Figure S3).

### 3.2. Activation of MAIT Cells by S. aureus-Infected DCs Is Enhanced by DC Production of IL-12

**Figure 2:**
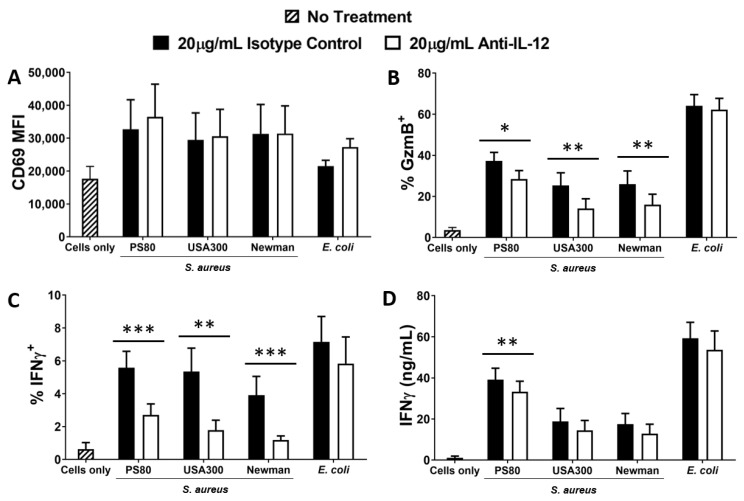
Inhibition of IL-12 results in reduced activation of MAIT cells by *S. aureus*-infected DCs. DCs (5 × 10^5^/mL) were infected with *S. aureus* (strains PS80, USA300, Newman) or *E. coli* (strain EC958) at MOI 10 for 3 h before elimination of extracellular bacteria by gentamicin treatment. DCs were then co-cultured with MAIT cells (5 × 10^5^/mL) for 24 h, with or without treatment with anti-IL-12p40 antibodies, or isotype controls (IgG1κ). Cells were treated with BFA for the final 4 h of culture. Expression of CD69 (**A**), Granzyme B (‘GzmB’) (**B**), and IFNγ (**C**) by MAIT cells were assessed by flow cytometry, while concentration of IFNγ (**D**) in culture supernatants was assessed by ELISA. Results are expressed as mean fluorescence intensity (MFI) of total live singlet CD3^+^ cells + SEM (**A**), mean % positive cells within total live singlet CD3^+^ cells + SEM (**B**,**C**), or mean concentration in culture supernatants + SEM (**D**). *n* = 5–7 DC donors per group, *n* = 8–14 MAIT cell donors per group. ‘Cells only’ refers to uninfected cultures. Statistical analysis by pairwise Wilcoxon signed rank test * *p* ≤ 0.05, ** *p* ≤ 0.01, *** *p* ≤ 0.001.

IL-12 is a key mediator of T cell activation, including MAIT cells, particularly for the secretion of IFNγ and cytotoxic mediators [56,57,58]. To establish the role of IL-12 in activating MAIT cells in response to *S. aureus* infection, DCs were infected with selected strains of *S. aureus* or *E. coli* for 3 h and, following elimination of bacteria by gentamicin treatment, resuspended in media supplemented with IL-12-neutralising Abs, and co-cultured with MAIT cells for 24 h. CD69 expression by MAIT cells was unaffected by IL-12 neutralisation (Figure 2A), but Granzyme B (Figure 2B) and IFNγ (Figure 2C) expression were significantly decreased when measured by flow cytometry. However, analysis of total culture supernatants revealed that actual levels of secreted IFNγ were less affected by IL-12 neutralisation (Figure 2D). The results indicate that IL-12 plays a significant but not exclusive role in the activation of MAIT cells in response to *S. aureus*-infected DCs. IL-12 neutralisation had no effect on *E. coli*-induced activation of MAIT cells which is consistent with previously published work [59]. Significant levels of IL-12 are produced by DCs following exposure to *S. aureus* (Figure S4A), but IL-18 is absent in DC cultures (Figure S4B); so unsurprisingly, IL-18-neutralising Abs had no effect on MAIT cell activation in this assay (Figure S4C).

### 3.3. Activation of MAIT Cells by S. aureus-Infected DCs Is Dependent on Cell–Cell Contact

**Figure 3:**
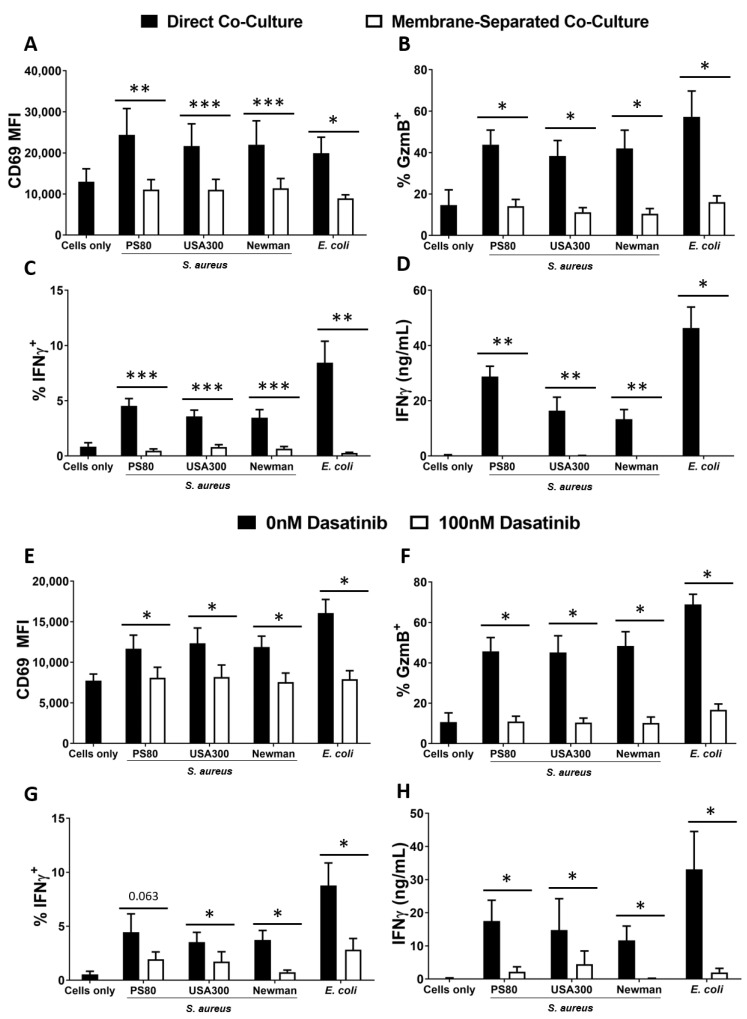
Blocking of cell–cell contact or of TCR signaling eliminates activation of MAIT cells by *S. aureus*-infected DCs**.** DCs (5 × 10^5^/mL) were infected with *S. aureus* (strains PS80, USA300, Newman) or *E. coli* (strain EC958) at MOI 10 for 3 h before elimination of extracellular bacteria by gentamicin treatment. DCs were then co-cultured with MAIT cells (5 × 10^5^/mL) for 24 h. Co-cultures were carried out in membrane-separated chambers (**A**–**D**) or treated with 100 nM Dasatinib (**E**–**H**). Cells were treated with BFA for the final 4 h of culture. Expression of CD69 (**A**,**E**), Granzyme B (‘GzmB’) (**B**,**F**) and IFNγ (**C**,**G**) by MAIT cells were assessed by flow cytometry, while concentration of IFNγ (**D**,**H**) in culture supernatants was assessed by ELISA. Results are expressed as mean fluorescence intensity (MFI) of total live singlet CD3^+^ cells + SEM (**A**,**E**), mean % positive cells within total live singlet CD3^+^ cells + SEM (**B**,**C**,**F**,**G**), or mean concentration in culture supernatants + SEM (**D**,**H**). *n* = 6–10 DC donors per group, *n* = 7–17 MAIT cell donors per group. ‘Cells only’ refers to uninfected cultures. Statistical analysis by pairwise Wilcoxon signed rank test. * *p* ≤ 0.05, ** *p* ≤ 0.01, *** *p* ≤ 0.001.

Alongside cytokine exchange, direct cell–cell contact also governs activation of conventional T cells by DCs. To determine the role of contact in the activation of MAIT cells during *S. aureus* infection, DCs were infected with selected strains of *S. aureus* or *E. coli* for 3 h and, following elimination of bacteria by gentamicin treatment, co-cultured with MAIT cells for 24 h in a multi-chamber system in which cell-cell contact was blocked by a 0.4µm membrane, without impeding exchange of secreted molecules. Activation of MAIT cells was significantly reduced as assessed by expression of CD69 (Figure 3A), Granzyme B (Figure 3B) and IFNγ (Figure 3C,D). CD69, Granzyme B and IFNγ levels were restored to those seen in MAIT cell-only cultures. These results indicate that, while MAIT cells have been shown elsewhere to respond to cytokines alone in an innate-like manner [58], they do not do so during *S. aureus* infection; instead, MAIT cell activation following *S. aureus* or *E. coli* infection is primarily dependent upon direct cell–cell contact.

To establish whether this contact was mediated via engagement of the TCR by the MHC-like molecule MR1 (shown previously to be a major route of MAIT cell activation [36]), we interrogated the TCR–MR1 axis by treatment of MAIT–DC co-cultures with blocking agents targeted against both molecules. First, to confirm the role of the TCR, co-cultures were treated with Dasatinib, a small molecule inhibitor of tyrosine kinases which has been found to specifically target TCR-related downstream signalling in T cells [54]. DCs were infected with selected strains of *S. aureus* or *E. coli* for 3 h and, following elimination of bacteria by gentamicin treatment, co-cultured with MAIT cells in the presence of Dasatinib. Expression of CD69, IFNγ and Granzyme B in response to both *S. aureus* and *E. coli* were all significantly decreased by Dasatinib treatment (Figure 3E–H). Although Dasatinib had some effect on DC activation (Figure S5), the magnitude of this response was much less than the overall effect observed on MAIT cell activation, which was of a similar magnitude to that observed when cell–cell contact was blocked.

We next assessed the effects of blocking the binding partner for the MAIT TCR, MR1. Previous studies have shown that when MR1 is blocked in MAIT cell co-cultures, activation is reduced, particularly at the earliest timepoints [57,60], suggesting that MR1-mediated activation of MAIT cells is both rapid and transitory, and likely replaced by other mechanisms such as IL-12 at later timepoints. We confirmed that MR1 is expressed by DCs and is unaffected by *S. aureus* infection (Figure S6A). To assess whether DC-expressed MR1 is a key binding partner for the MAIT cell TCR in *S. aureus* infection, MAIT–DC co-cultures were treated with MR1-blocking Abs and analysed at multiple timepoints.

**Figure 4:**
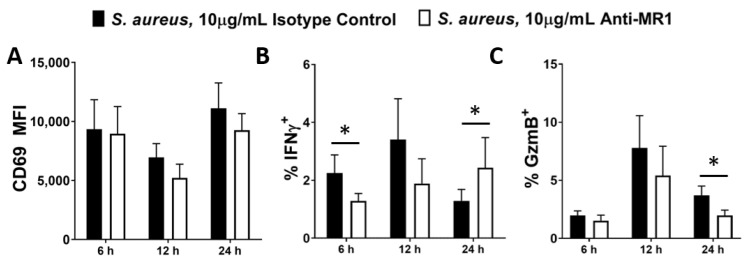
Inhibition of MR1 during *S. aureus* infection delays MAIT cell activation. DCs (5 × 10^5^/mL) were infected with *S. aureus* (strain PS80) at MOI 10 for 3 h before elimination of extracellular bacteria by gentamicin treatment. DCs were then co-cultured with uninfected MAIT cells (5 × 10^5^/mL) for 6–24 h, with or without treatment with anti-MR1 antibodies or the isotype control (IgG2aκ). Cells were treated with BFA for the final 4 h of culture. Expression of CD69 (**A**), IFNγ (**B**) and Granzyme B (‘GzmB’) (**C**) by MAIT cells were assessed by flow cytometry. Results are expressed as mean fluorescence intensity (MFI) of total live singlet CD3^+^ cells + SEM (**A**) or mean % positive cells within total live singlet CD3^+^ cells + SEM (**B**,**C**). *n* = 3–6 DC donors per group, *n* = 6 MAIT cell donors per group. ‘Cells only’ refers to uninfected cultures. Statistical analysis by pairwise Wilcoxon signed rank test, comparing anti-MR1-treated cultures to isotype controls. * *p* ≤ 0.05.

MR1 blockade non-significantly reduces CD69 (Figure 4A) and significantly reduces Granzyme B (Figure 4C) expression by MAIT cells in response to *S. aureus*, while it appears to delay IFNγ expression. In the absence of MR1 blockade, IFNγ peaks at 12 h (Figure 4B), before declining at 24 h. However, the peak for IFNγ expression in MR1-blockaded cultures does not occur until 24 h or afterwards. At 6 h, MR1-blockaded cells display significantly reduced IFNγ production, but at 24 h, IFNγ production in these cells has recovered, displaying the delayed peak of a slower response (Figure 4B). IFNγ production was therefore not completely eliminated by MR1 blockade, but instead it was slowed down, which may be of paramount importance in an acute infection context such as *S. aureus* bacteraemia. Combined treatment with anti-MR1 and anti-IL-12 antibodies appears to further reduce Granzyme B expression at 12 h, (Figure S7) suggesting that IL-12 and MR1 act independently to induce production of this cytotoxic mediator. MR1 blockade significantly reduced *E. coli*-induced activation of MAIT cells (Figure S6B,C) as has previously been reported [33,59].

In conclusion, these results show that *S. aureus*-induced responses by MAIT cells are controlled both by MR1 and by IL-12. Binding of MR1 by the MAIT TCR induces IFNγ within 6 h of exposure to *S. aureus*-infected DCs, while CD69 and Granzyme B responses, also induced by MR1, become detectable after 6 h. IL-12 released by infected DCs likely acts to enhance expression of CD69, IFNγ and Granzyme B by MAIT cells, further arming these cells for tackling *S. aureus* infection.

### 3.4. MAIT Cells Mediate Reduced Intracellular Survival of S. aureus

**Figure 5:**
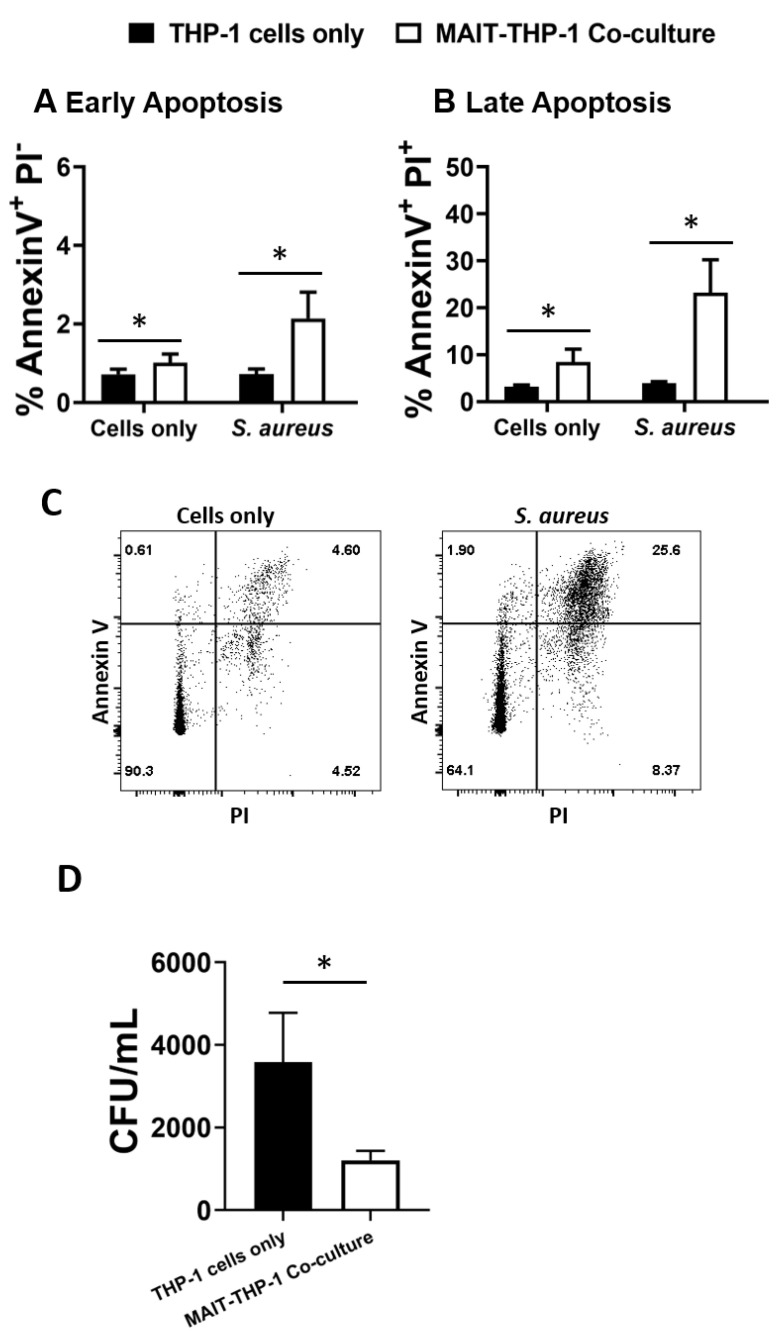
MAIT cells prevent *S. aureus* intracellular survival within THP-1 cells. THP-1 cells (5 × 10^5^/mL) were infected with *S. aureus* (strain PS80) at MOI 1 for 3 h before elimination of extracellular bacteria by gentamicin treatment. THP-1 cells were then either cultured alone or co-cultured with uninfected MAIT cells (5 × 10^5^/mL) for 24 h, after which cells were either collected for analysis of apoptosis by Annexin V and PI staining (**A**–**C**) or washed in PBS and lysed in 0.1% Triton X-100 for 10 min (**D**). Lysates were plated on TSA plates and incubated overnight. CFUs were enumerated the following day, and CFU/mL of original wells calculated. Results are expressed as mean percentage of apoptotic cells + SEM (**A**,**B**) or mean CFU/mL + SEM (**D**). Representative FACS plots are shown (**C**). *n* = 5–6 MAIT cell donors per group. ‘Cells only’ refers to uninfected cultures. Statistical analysis by pairwise Wilcoxon signed rank. * *p* ≤ 0.05.

The adoption of a cytotoxic phenotype by MAIT cells in response to *S. aureus* infection raises the question of whether this represents an immune defence mechanism that can be deployed to control *S. aureus,* given the potential for *S. aureus* to maintain an intracellular lifestyle to promote persistence and dissemination [45,46,47]. THP-1 cells (5 × 10^5^ cells/mL), which have previously been shown to be a receptive intracellular niche for *S. aureus* [61,62,63], were infected with *S. aureus* strain PS80 (a strain with a propensity for intracellular survival [46,64]) for 3 h before gentamicin treatment to eliminate extracellular bacteria. MAIT cells (5 × 10^5^ cells/mL) were then co-cultured with infected THP-1 cells for 24 h, alongside MAIT cell-free THP-1 cultures. After 24 h, cells were collected and stained with anti-Annexin V Abs and PI, or were washed in PBS and lysed with 0.1% Triton X-100 to enumerate intracellular bacteria. Annexin V staining revealed that MAIT cell co-culture significantly elevates the apoptosis of *S. aureus*-infected THP-1 cells (Figure 5A–C). Using PI to differentiate early and late apoptotic cells, we determined that most apoptotic cells were late apoptotic by 24 h. Analysis of CFUs from cell lysates showed a significant and profound reduction of *S. aureus* persistence in co-cultured THP-1 cells compared to cells cultured without MAIT cells (Figure 5D). These results show clearly that MAIT cells mediate the killing of intracellular *S. aureus* through cytotoxic mechanisms, and could hypothetically make major contributions to the clearance of *S. aureus* infection within the bloodstream.

## 4. Discussion

A reduction in circulating MAIT cells is associated with increased susceptibility to a variety of viral and bacterial infections [36], including sepsis [41], and in particular nosocomial infections [41]. Given the dominance of *S. aureus* as a causative organism of both bacteraemia [65,66] and nosocomial infections [67], a better understanding of the role played by circulating MAIT cells in response to *S. aureus* is warranted. If MAIT cell deficiencies are linked to *S. aureus* susceptibility, then activation or even adoptive transfer of MAIT cells in *S. aureus* patients might then be considered as promising new treatment strategies, while vaccination might be expected to see greater success if MAIT cells are targeted. Here we established a model for the activation of bloodstream-derived MAIT cells in response to *S. aureus*. Our assays reveal that MR1, expressed by *S. aureus*-infected DCs, mediates MAIT cell activation via the TCR, resulting in rapid IFNγ production. MR1 also mediates Granzyme B accumulation in activated MAIT cells, while the innate cytokine IL-12 acts to enhance production of both IFNγ and Granzyme B. After 24 h, activated MAIT cells produce substantial levels of both the key anti-*S. aureus* cytokine IFNγ and the key cytotoxic mediator Granzyme B. These MAIT cells mediate the killing of THP-1 cells infected with *S. aureus*, with the result that *S. aureus* persistence is strikingly reduced in cultures with MAIT cells. These studies significantly advance our understanding of the breadth of MAIT cell responses to *S. aureus*, with unique insights into their mechanism of activation, cytotoxic responses, and importantly, potency for killing invasive *S. aureus*.

While MAIT cells in mice are typically scarce, their absence is nonetheless associated with susceptibility to a number of invasive pathogens [34,35,36,37,38,68]; it is natural to wonder if the much larger human MAIT cell compartment might be even more important in human immune responses to infection. In light of repeated failures to transfer protective immunity achieved with *S. aureus* vaccines in mice to human test subjects, it is essential that we develop new assay systems to identify immunological phenomena that may be underrepresented in murine system. MAIT cells, whose frequency is so starkly different between mice and humans, are an obvious case in point.

Human MAIT cells have previously been demonstrated to produce IFNγ [39,69,70,71,72] following exposure to *S. aureus* or bacterial supernatants. However, these studies have left open the question of how these responses are induced. In addition, the possibility that MAIT cells might also tackle *S. aureus* infections by cytotoxicity has not been addressed. MAIT cells in the bloodstream, with their capacity for rapid MHC-independent activation [55], are ideally suited for rapid elimination of *S. aureus* and the prevention of its dissemination through the bloodstream to distal bodily tissues. The potential for *S. aureus* superantigens to activate MAIT cells for rapid and deleterious inflammatory cytokine responses has been noted [43], and if cytokine production by MAIT cells during *S. aureus* infection is actually a harmful part of toxic shock syndrome, then this indicates that caution should be exercised if centring innovative clinical solutions around these cells. However, in these superantigen studies, MAIT cell hyperactivation occurred only after exposure to innate cytokines produced by other cells. Our study employed *S. aureus* strains with disparate superantigen production levels; PS80 is reported to have reduced superantigen expression [73], while USA300 expresses a broad repertoire [74]. In our assays, MAIT cells directly exposed to *S. aureus* bacteria were not activated, suggesting that superantigens alone are not sufficient for robust MAIT cell activation.

Having confirmed the secretion of IFNγ and Granzyme B by MAIT cells in co-cultures with *S. aureus*-infected DCs, it was important to assess how these responses are mobilised. We found that both the innate cytokine IL-12 and the TCR-MR1 axis played a part. IL-12 treatment alone does not induce production of IFNγ by MAIT cells [57]. Where TCR-independent IFNγ expression has been observed (such as during in vitro viral infection), IL-12 neutralisation has no effect on it [58]; instead, it appears that a cocktail of cytokines is required to induce IFNγ expression in these circumstances, with IL-18 a critical constituent of such cocktails [57,58]. Similarly, IL-15, another key innate cytokine, has been found to trigger IFNγ production by MAIT cells in combination with IL-18 [75]. These studies suggest that IL-18 is the key ingredient for TCR-independent activation of MAIT cells. IL-18, while crucially important for the activation of MAIT cells during infections by viruses [58,76], including SARS-CoV-2 [40], played no role in the activation of MAIT cells in our assay where *S. aureus* failed to induce any IL-18 production by DCs. In the absence of IL-18 in response to *S. aureus*, IL-12 appears to play a secondary role in our assays; neutralising it weakens activation but does not abolish it. Thus, while our study confirms that IL-12, secreted by *S. aureus*-infected DCs, plays a role in mediating MAIT cell activation in response to *S. aureus*, our study also confirms that this role is not exclusive.

Instead, the TCR-MR1 axis was found to be crucial for MAIT cell activation. Activation of MR1, the key MAIT TCR ligand, is essential for MAIT cell responses to *Fusobacterium nucleatum* [77], *M. bovis* BCG [35], and frequently *E. coli*, where MR1 blockade has the strongest effect on MAIT cell activation at early timepoints. In co-cultures of MAIT cells with THP-1 cells exposed to formaldehyde- or paraformaldehyde-fixed *E. coli*, the negative effects of MR1 blockade on MAIT cell activation go from total or near-total at earlier timepoints, to only partial at 20–24 h [57,60]. Assays using *E. coli*-exposed primary monocytes [36,78], macrophages [59] and B cells [33] have shown similar results of MR1 blockade. We recapitulated these results for *E. coli*-infected DCs, and furthermore showed that the same is at least partially true for *S. aureus*: MR1 blockade significantly reduces MAIT cell IFNγ production in response to this pathogen at 6 h but not at 24 h. In our assay, a picture emerged where *S. aureus*-induced IFNγ production commenced rapidly at 6 h in response to MR1 binding and continued to rise thereafter, but by 24 h was chiefly controlled by the influence of IL-12, such that MR1 blockade no longer held it back. Clearly, IL-12 neutralisation had a significant dampening effect. Granzyme B production on the other hand was undetectable at 6 h and only gradually increased over 12–24 h under the influence of both MR1 and IL-12, such that blocking either of these signals significantly reduced Granzyme B production. These findings emphasised the importance of MR1 binding of the MAIT TCR, particularly at the earliest timepoints, suggesting that riboflavin intermediates known to be produced by staphylococcal metabolism [79] could be a major early activation signal for MAIT cells in the bloodstream. However, it is worth noting that TCR blocking had a profound dampening effect on activation at 24 h when MR1 blocking did not. While this may be an artefact of the collateral effects of Dasatinib on DCs, it may also suggest that another binding partner for the MAIT TCR may have had a role to play in MAIT cell activation.

Crucially, this is the first report of MAIT cell cytotoxicity marshalled against intracellular *S. aureus*. Co-culture with *S. aureus*-infected DCs induced MAIT cell degranulation (measured as CD107a expression), and also production of Granzyme B, a key cytolytic mediator that could be involved in the containment of intracellular *S. aureus* infections. Granzyme B has substantial ability to kill *S. aureus* directly in vitro [80], and the blocking of neutrophil-expressed Granzyme B prevents the neutrophil-mediated clearance of MRSA in infected mice [81]. *S. aureus* infection of PBMCs results in upregulation of the degranulation marker CD107a in other cell types [72], supporting the notion that activation of cytotoxic responses against *S. aureus*-infected cells can occur in the systemic circulation. MAIT cells efficiently mediate the death of cells infected with a number of bacteria, including *E. coli* [82,83,84], *Shigella flexneri* [82], *H. pylori* [85], *Mycobacterium smegmatis* [86] and *Haemophilus influenzae* [87], through the expression of Granzyme B. However, while a number of cell types harbouring intracellular bacteria have been shown to be lysed by MAIT cells [82,83,84,85,86,87], DCs are, to the best of our knowledge, not among them. We demonstrated, at least in the initial 24 h, that cytolysis of *S. aureus*- and *E. coli*-infected DCs was minimal, in spite of the clear increase in cytotoxic potential shown by Granzyme B expression. This is likely because activated DCs express high levels of serine protease inhibitor (serpin), which specifically protects them from the effects of Granzyme B [88]. This resistance is enhanced by co-culture with T_h_1 cells [88]; MAIT cells in our assays showed a pronounced IFNγ-producing phenotype, and it is therefore quite possible that these cells may also have mediated enhanced DC resistance to Granzyme B. Our experiments instead employed THP-1 cells to assess MAIT cell cytotoxicity, being that these cells are notable intracellular niches for *S. aureus* survival [61,62], and we found that MAIT cell co-culture significantly heightened apoptosis of infected cells, resulting in a roughly threefold reduction in the persistence of intracellular *S. aureus*. It is worth noting that this may not exclusively be a result of cytotoxicity, but also of the MAIT cells’ substantial IFNγ expression. Among a number of specific roles in fighting *S. aureus* infection, including macrophage activation [17] and neutrophil survival [21], IFNγ frequently licences infected cells to overcome blocks on intracellular killing; for example, macrophages kill intracellular *S. aureus* much more efficiently if treated with IFNγ [20], and MAIT cells specifically have been shown to limit intracellular growth of *M. bovis* in infected macrophages via IFNγ secretion [35]. However, we believe that the profound cytotoxic effects of MAIT cells, demonstrated by Annexin V staining data, heavily imply that this mechanism takes precedence over IFNγ-mediated *S. aureus* killing. Notably, both of these potential mechanisms—*S. aureus* elimination mediated either by cytotoxic mediators or by IFNγ—are likely to be dependent on IL-12 secretion by infected cells and direct TCR binding by MR1, in light of our findings that Granzyme B and IFNγ rely on both of these signals for deployment.

It has been suggested that in vitro-expanded MAIT cells could be administered to patients suffering bacteraemia and other severe infections in order to alleviate both primary infections and sequelae [89]. The data presented here add to this case, and add *S. aureus* to the list of potential targets. Their broad effector functions have also provoked the suggestion of targeting MAIT cells for vaccination [90]. In a specific murine vaccination trial, clearance of *L. longbeachae* was enhanced in mice following immunization with the key MR1 ligand 5-OP-RU in combination with IL-23, highlighting a potential role for targeting MAIT cells in vaccination trials [91]. During controlled infection of human volunteers with *S. enterica* serovar Paratyphi A, MAIT cells undergo clonal expansion, and expanded clonotypes show greater in vitro activation, suggesting a memory-like phenomenon [92]. Most recently, MR1 deficiency in mice resulted in defective responses by total CD8^+^ T cells to the ChAdOx1 SARS-CoV-2 vaccine [93]. These studies demonstrate that as we learn more about MAIT cells, their potential as targets for advanced next generation *S. aureus* treatments becomes clearer.

Taken together, these findings reveal the important effector responses of MAIT cells in response to *S. aureus*, and clarify the precise mechanisms and time course of that response. MAIT cells have unique characteristics that make them attractive tools for novel *S. aureus* therapies, and their powerful contributions to both IFNγ and cytotoxic responses have clear relevance for defence against this devastating infection, raising the possibility of administering them as treatments during infection, or of targeting them in next generation vaccine trials. The hunt for an *S. aureus* vaccine requires unconventional thinking, and these unconventional T cells should no longer be overlooked.

## Supplementary Materials

The following are available online at https://www.mdpi.com/article/10.3390/microorganisms10010148/s1, Figure S1: The majority of human blood-derived MAIT cells are CD8^+^CD4^−^ and CD161^hi^. Figure S2: Human blood-derived MAIT cells in co-culture with *S. aureus*-infected DCs express TNF. Figure S3: Activated MAIT cells do not directly kill DCs at 24 h. Figure S4: *S. aureus* induces IL-12 secretion and not IL-18 secretion by DCs. Figure S5: Treatment with Dasatinib has minor effects on DC activation and no effect on viability. Figure S6: *S. aureus*-infected DCs express MR1, and inhibition of MR1 during *E. coli* infection reduces MAIT cell activation. Figure S7: Combined blocking of MR1 and IL-12 further decreases Granzyme B expression by MAIT cells in response to *S. aureus*-infected DCs at 12 h.
