# Supplementary material for: Human MAIT Cells Respond to Staphylococcus aureus with Enhanced Anti-Bacterial Activity
Source: Microorganisms. 2022 Jan 12;10(1):148. doi: 10.3390/microorganisms10010148 (PMC8778732; doi:10.3390/microorganisms10010148)
Supplement: Supplementary file 1 [file microorganisms-10-00148-s001.zip › microorganisms-1511822-Supplemental.pdf]

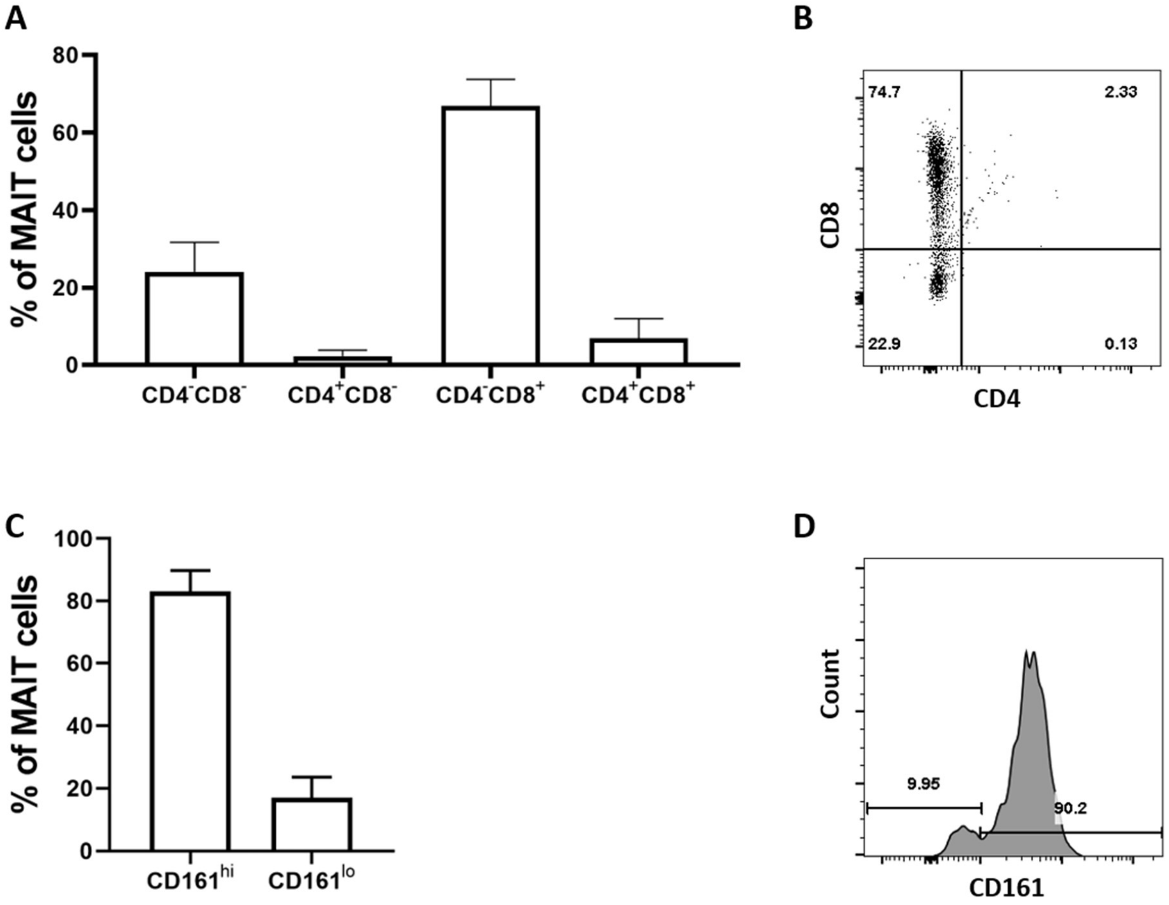

Figure S1. The majority of human blood-derived MAIT cells are $CD8^{+}CD4^{-}$ and $CD161^{hi}$. MAIT cells were expanded *in vitro* by treatment with 5-A-RU and methylglyoxal for 10-12 days and purified by MACS. Cells were then stained with an MR1 tetramer and with antibodies for CD4 and CD8 (**A**) and CD161 (**C**) and analysed by FACS. Results are expressed as mean % of total live singlet MR1-tetramer-positive cells + SEM. Representative FACS plots are shown (**B**, **D**). n=5 MAIT cell donors per group.

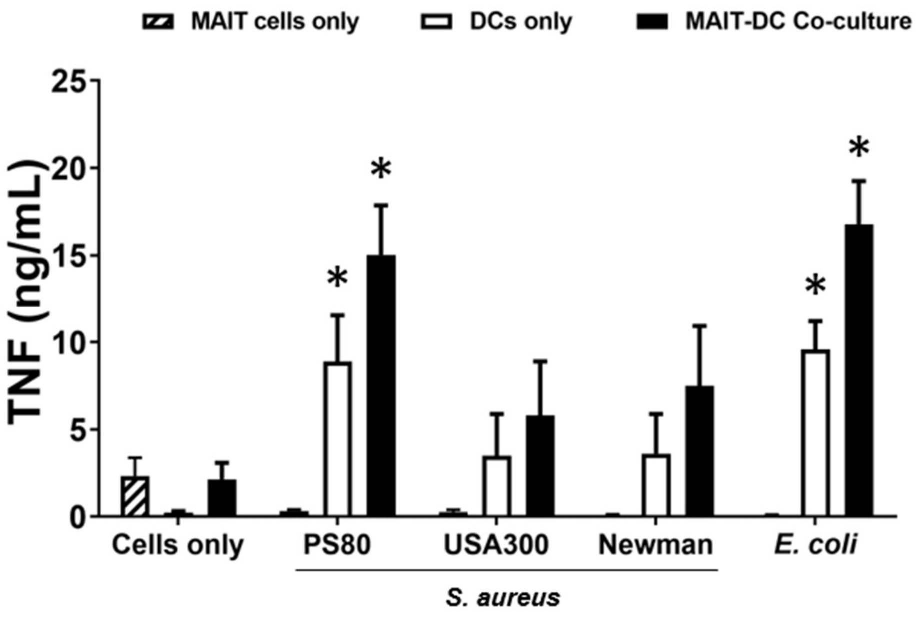

Figure S2. Human blood-derived MAIT cells in co-culture with *S. aureus*-infected DCs express TNF. MAIT cells and DCs ($5x10^5$ cells/mL) were infected with *S. aureus* (strains PS80, USA300, Newman) and *E. coli* (strain EC958) at MOI 10 for 3 h before elimination of extracellular bacteria by gentamicin treatment. DCs were then co-cultured with uninfected MAIT cells ($5x10^5$/mL) for 24 h. Concentration of TNF in culture supernatants was assessed by ELISA. Results are expressed as mean concentration in culture supernatants + SEM. n=6 DC donors per group, n=6 MAIT cell donors per group. 'Cells only' refers to uninfected cultures. Statistical analysis by pairwise Wilcoxon signed rank test, with all columns compared directly to cells only. *$p \leq 0.05$.

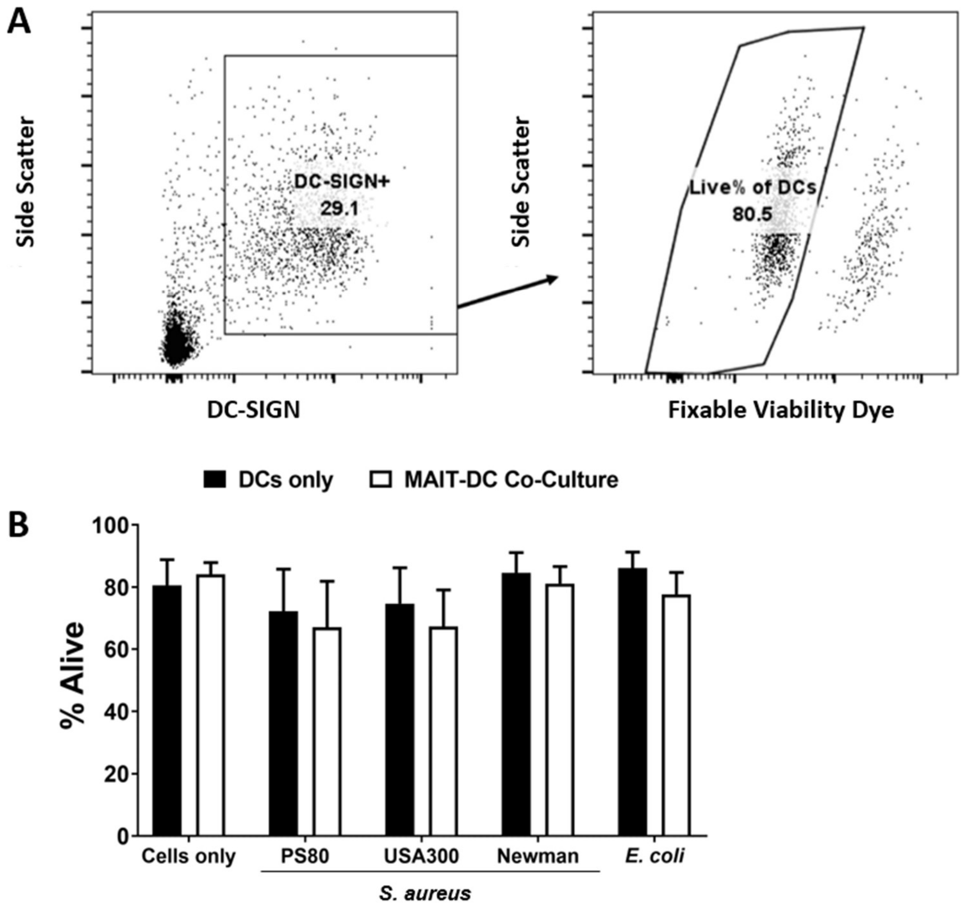

Figure S3. Activated MAIT cells do not directly kill DCs at 24 h. DCs (5x10^5/mL) were infected with *S. aureus* (strains PS80, USA300, Newman) or *E. coli* (strain EC958) at MOI 10 for 3 h before elimination of extracellular bacteria by gentamicin treatment. DCs were then co-cultured with MAIT cells (5x10^5/mL) for 24 h. DC viability was assessed by flow cytometry. Representative FACS plots for PS80 infection are shown (**A**). Results are expressed as mean % live cells within total singlet DC-SIGN$^+$ cells + SEM (**B**). n=7 DC donors per group, n=7 MAIT cell donors per group. 'Cells only' refers to uninfected cultures. Statistical analysis by pairwise Wilcoxon signed rank test.

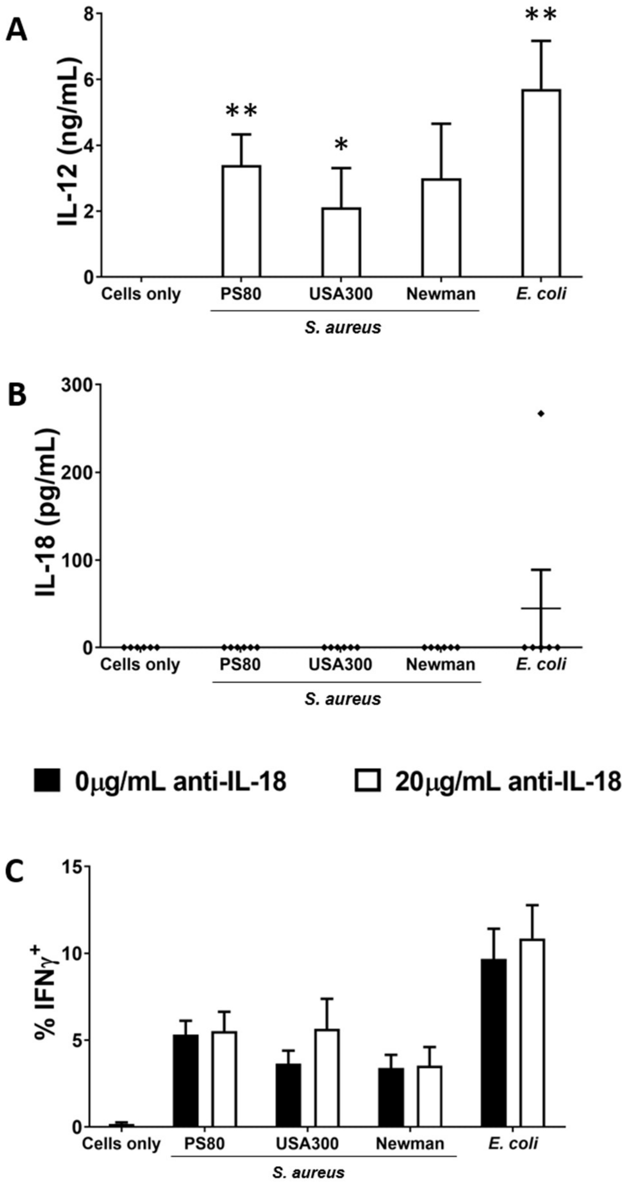

Figure S4. *S. aureus* induces IL-12 secretion and not IL-18 secretion by DCs. DCs ($5x10^5$/mL) were infected with *S. aureus* (strains PS80, USA300, Newman) or *E. coli* (strain EC958) at MOI 10 for 3 h before elimination of extracellular bacteria by gentamicin treatment. DCs were then co-cultured with uninfected MAIT cells ($5x10^5$/mL) for 24 h. Selected co-cultures were treated with 20µg/mL IL-18-neutralising Abs (**C**). Cells were treated with BFA for the final 4 h of culture. Concentration of IL-12 (**A**) and IL-18 (**B**) in culture supernatants was assessed by ELISA, and expression of IFNγ by MAIT cells measured by flow cytometry (**C**). Results are expressed as mean concentration in culture supernatants + SEM (**A**), as individual values ± SEM (**B**), or mean % IFNγ$^+$ cells within total live singlet CD3$^+$ cells + SEM (**C**). n=3-7 DC donors per group, n=6-14 MAIT cell donors per group. 'Cells only' refers to uninfected cultures. Statistical analysis by pairwise Wilcoxon signed rank test, with columns in **A** and **B** compared directly to cells only. *$p \leq 0.05$.; **$p \leq 0.01$

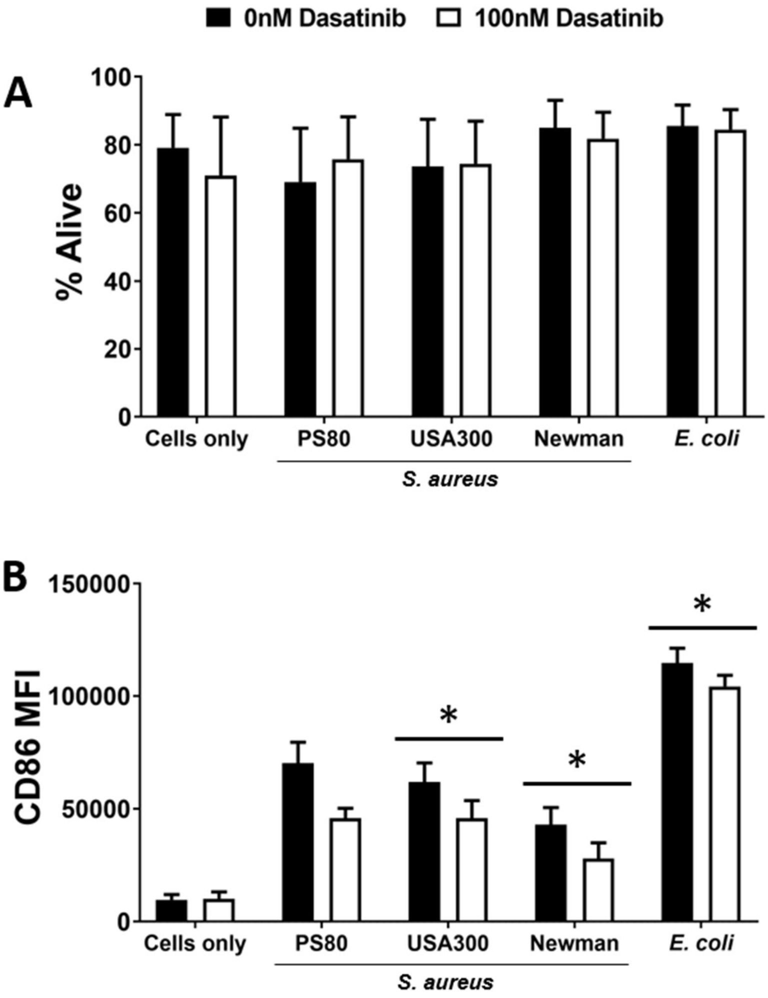

Figure S5. Treatment with Dasatinib has minor effects on DC activation and no effect on viability. DCs (5x10^5/mL) were infected with *S. aureus* (strains PS80, USA300, Newman) or *E. coli* (strain EC958) at MOI 10 for 3 h before elimination of extracellular bacteria by gentamicin treatment. DCs were then treated with the tyrosine kinase inhibitor molecule Dasatinib and cultured for 24 h. DC viability (**A**) and CD86 expression (**B**) were assessed by flow cytometry. Results are expressed as mean % live cells within total singlet DC-SIGN$^+$ cells + SEM (**A**) or mean fluorescence intensity (MFI) of total live singlet DCs + SEM (**B**). n=4-6 DC donors per group. 'Cells only' refers to uninfected cultures. Statistical analysis by pairwise Wilcoxon signed rank test. *p ≤ 0.05.

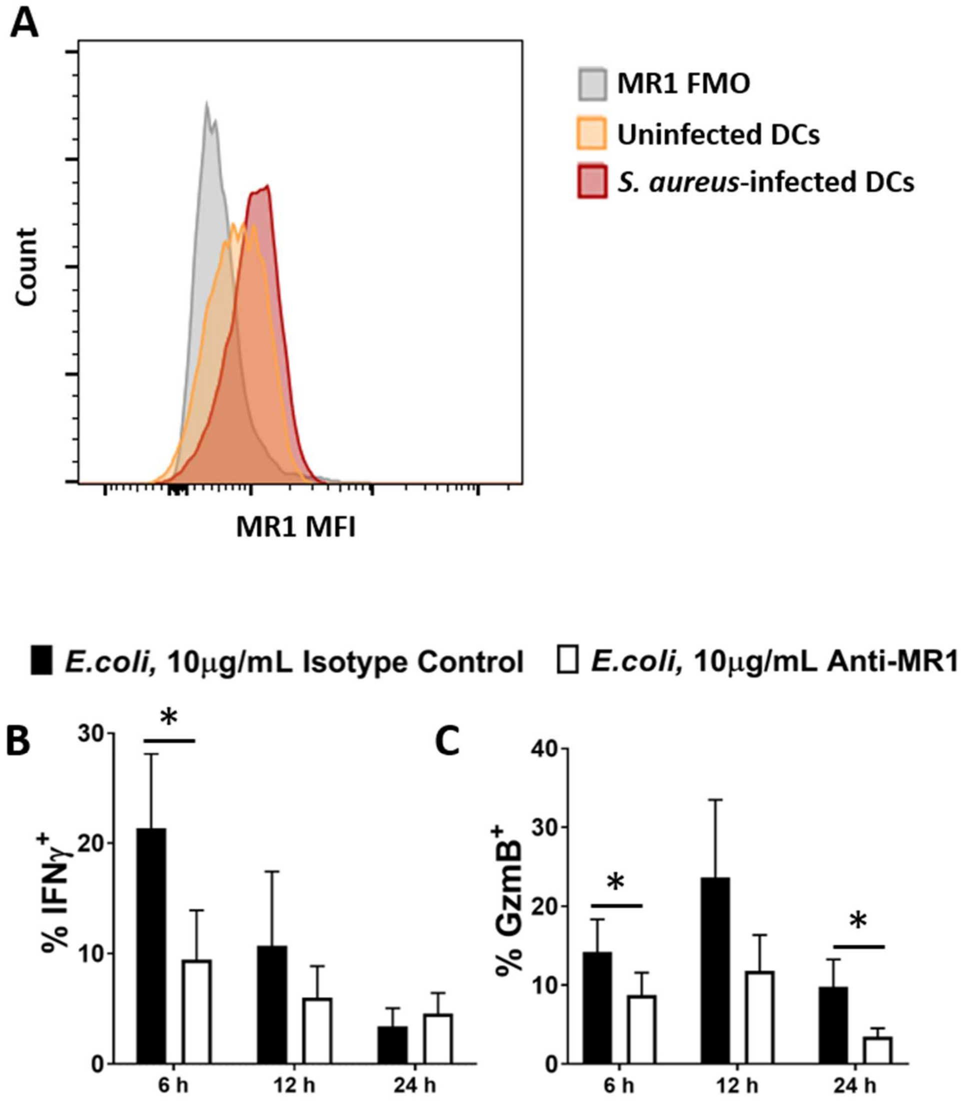

Figure S6. ***S. aureus*****-infected DCs express MR1, and inhibition of MR1 during** ***E. coli*** **infection reduces MAIT cell activation.** DCs ($5x10^5$/mL) were infected with *S. aureus* (strain PS80) at MOI 10 for 3 h before elimination of extracellular bacteria by gentamicin treatment. MR1 expression on the DCs was then analysed by FACS (representative FACS plot shown, comparing FMO to stained cells, (**A**)). DCs ($5x10^5$/mL) were infected with *E.coli* (strain EC958) at MOI 10 for 3 h before elimination of extracellular bacteria by gentamicin treatment and then co-cultured with MAIT cells ($5x10^5$/mL) for 6-24 h, with or without treatment with anti-MR1 antibodies or the isotype control (IgG2aκ). Co-cultured cells were treated with BFA for the final 4 h of culture. Expression of IFNγ (**B**) and Granzyme B (**C**) by MAIT cells were assessed by flow cytometry. Results are expressed as mean % positive cells within total live singlet $CD3^+$ cells + SEM. n=3-6 DC donors per group, n=5-7 MAIT cell donors per group. Statistical analysis by pairwise Wilcoxon signed rank test, comparing anti-MR1-treated cultures to isotype controls. *p ≤0.05.

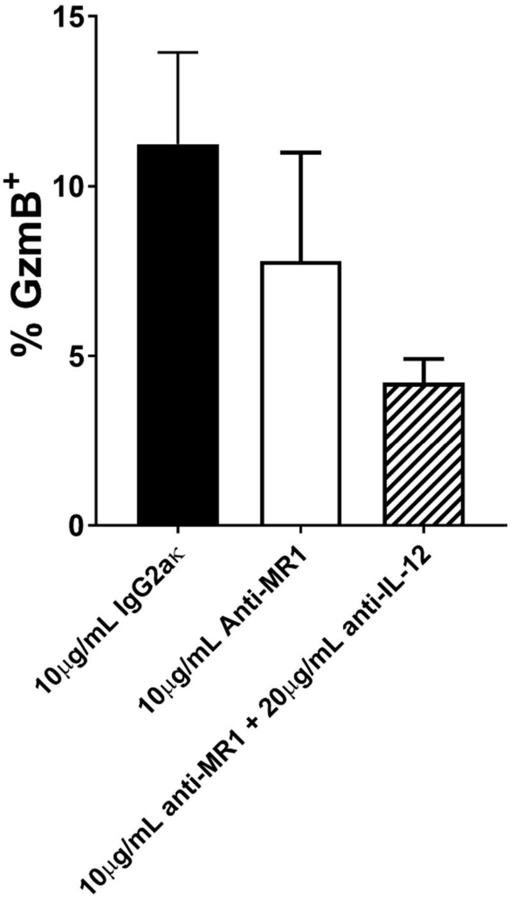

Figure S7. Combined blocking of MR1 and IL-12 further decreases Granzyme B expression by MAIT cells in response to *S. aureus*-infected DCs at 12 h. DCs ($5x10^5$/mL) were infected with *S. aureus* (strain PS80) at MOI 10 for 3 h before elimination of extracellular bacteria by gentamicin treatment. DCs were then co-cultured with uninfected MAIT cells ($5x10^5$/mL) for 12 h. Selected co-cultures were treated with 10µg/mL MR1-blocking Abs and 20µg/mL IL-12-neutralising Abs. Cells were treated with BFA for the final 4 h of culture. Expression of Granzyme B by MAIT cells was assessed by flow cytometry. Results are expressed as mean % positive cells within total live singlet $CD3^+$ cells + SEM. n=4 DC donors per group, n=4 MAIT cell donors per group.
